# Parenting Information on Social Media: Systematic Literature Review

**DOI:** 10.2196/55372

**Published:** 2024-10-23

**Authors:** Ellen Mertens, Guoquan Ye, Emma Beuckels, Liselot Hudders

**Affiliations:** 1 Department of Communication Sciences Ghent University Ghent Belgium; 2 School of Journalism and Communication Xiamen University Xiamen China

**Keywords:** parenting, social media, parenting information, systematic literature review, bibliometric literature review, thematic analysis

## Abstract

**Background:**

Social media has become extremely popular among parents to seek parenting information. Despite the increasing academic attention to the topic, studies are scattered across various disciplines. Therefore, this study broadens the scope of the existing reviews by transcending narrow academic subdomains and including all relevant research insights related to parents’ information seeking on social media and its consequent effects.

**Objective:**

The aims of this systematic literature review were to (1) identify influential journals and scholars in the field; (2) examine the thematic evolution of research on parenting and social media; and (3) pinpoint research gaps, providing recommendations for future exploration.

**Methods:**

On the basis of a criteria for identifying scholarly publications, we selected 338 studies for this systematic literature review. We adopted a bibliometric analysis combined with a content thematic analysis to obtain data-driven insights with a profound understanding of the predominant themes in the realm of parenting and social media.

**Results:**

The analysis revealed a significant increase in research on parenting and social media since 2015, especially in the medical domain. The studies in our review spanned 232 different research fields, and the most prolific journal was *JMIR Pediatrics and Parenting*. The thematic analysis identified 4 emerging research themes in the studies: parenting motivations to seek information, nature of parenting content on social media, impact of parenting content, and interventions for parents on social media.

**Conclusions:**

This study provides critical insights into the current research landscape of parenting and social media. The identified themes, research gaps, and future research recommendations provide a foundation for future studies, guiding researchers toward valuable areas for exploration.

## Introduction

### Background

The experience of transitioning into parenthood often elicits a feeling of being overwhelmed [1], through which parents may encounter an intricate transformation of their identity [2]. Achieving a balance among their parental obligations, roles as partners, and individual identities presents a difficult challenge [3]. In addition, they are confronted with social pressures and societal norms surrounding parenthood [3]. In today’s digital era, social media plays an important role in how parents manage everyday issues and decisions [4,5]. This comes as no surprise given the massive popularity of social media, with 4.89 billion users worldwide in 2023 [6]. The largest group of users is aged 25 to 34 years [7], which corresponds to the age group of many young parents [8]. Research has demonstrated that parents actively seek online social support and parenting information [5,9,10]. Previous generations have often relied on family and close friends for parenting information, whereas today’s parents depend heavily on social media, where they share information and experiences with like-minded others [10]. Obtaining this informational and emotional support empowers parents to feel prepared and confident in their new roles, easing their transition into parenthood [11].

The impact of social media on parents has been studied across various fields, such as health sciences, communication, and pedagogic research [12,13]. For example, within the field of persuasive communication, *momfluencers* (a portmanteau of the words “mom” and “influencer”) have been demonstrated to generate feelings of support and understanding among parents but can also cause stress, lower parental efficacy, and anxiety [8,14,15]. Within the health information domain, Chan and Chen [16] found that social media represents an effective source for improving maternal health, mental health, and pregnancy knowledge. Hence, various individual studies from different research domains have made significant contributions by examining parents’ information seeking on social media and its consequent impact on their lives and decision-making [13,17,18].

While considerable research exists at the intersection of parenthood and social media, comprehensive review papers that summarize collective insights in this domain are extremely limited. Within the overall field, we identified 6 review papers that addressed subtopics related to that of this study. In total, 2 papers evaluated specific social media interventions for parents [16,19], 2 focused on health information for parents [10,20], and 2 addressed subdomains such as a target group (eg, military families [21]) or a specific variable (eg, family connectedness [22]). Moreover, it is crucial to highlight that a significant proportion of studies that have explored the impact of social media on contemporary parenting are predominantly situated within the realm of medical research [10,19]. However, despite the notable interest of media and communication scholars in this subject [15,22], their research remains fragmented. Consequently, a comprehensive systematic review of parenting information disseminated through social media from a media and communication perspective can significantly enhance our understanding of this field.

To address this gap, this study broadens the scope of existing reviews by transcending narrow academic subdomains and including all relevant research insights related to parents’ information seeking on social media and its effects. Using both bibliometric and content thematic analyses, our approach combined objectivity and data-driven insights with an understanding of key themes in the realm of parenting and social media [23,24]. This allowed us to provide an overview of the research, detect patterns, delineate topics, and identify knowledge gaps [24]. The main objectives of this study were to (1) identify the journals and scholars who are actively involved in, contribute significantly to, and exert the most influence in the field; (2) examine the themes explored in existing research on parenting and social media and how they have evolved over time; and (3) highlight current research gaps and provide recommendations for further exploration in this domain.

### Literature Review

Several review papers in the field of parental information seeking on social media are pertinent to our study. Among them, 6 were found to align with our focus on social media, whereas 1 fell outside due to its focus on the internet in general. Nevertheless, the findings of the aforementioned systematic literature review were deemed relevant and, therefore, are briefly discussed as follows. Plantin and Daneback [25] consolidated research on how parents use the internet to access child-, health-, and family-related support and information, as well as how professionals use it to offer support and information to parents. They concluded that parents’ tendency to seek online information is explained by the desire to seek support and information. They argued that this is mostly driven by the anonymous nature of online information seeking and its round-the-clock accessibility. For professionals, key benefits include cost-efficiency and reaching a large audience. However, the study was published 14 years ago, and the studies in the authors’ sample were mostly conducted during the Web 1.0 era, when the internet predominantly consisted of static, informational websites. Since then, parents’ online experiences have significantly evolved with increased interactivity and the widespread use of social media. Therefore, we considered it important to reassess and synthesize the research on how parents engage with the wealth of real-time information on social media, which often includes personalized, user-generated advice, creating collaborative, global communities of parents.

The aforementioned 6 review studies that focused on social media had specific thematic focuses on interventions [19]; particular subdomains of parenting information, such as health information [10]; specific target groups, such as military families [21]; or demarcated variables, such as family connectedness [22]. The following paragraphs discuss these 6 reviews on social media and parenting.

Of the reviews, 2 evaluated the effectiveness of social media interventions for parents. First, Hamm et al [19] conducted a systematic review in 2014 analyzing 25 studies on the use and effectiveness of social media in child health interventions. Their review provided insights into how social media is used in interventions promoting child health, such as encouraging healthy eating and exercise among children and adolescents [19]. The authors highlighted that social media interventions that aim to improve children’s health predominantly focus on adolescents rather than on children and parents [19]. In addition, they evaluated the effectiveness of these interventions and the factors driving their success [19]. Despite reported benefits from interventions using discussion forums, no studies using such forums achieved significant health outcomes [19]. Second, Chan and Chen [16] conducted a meta-analysis of 16 papers on the effectiveness of social media and mobile apps in pregnancy care. In contrast to the study by Hamm et al [19], their review found that interventions using mobile apps and social media in the context of pregnancy care were effective, with moderate to large effect sizes in maternal health, mental health, and pregnancy knowledge [16].

Furthermore, 2 studies focused on specific target groups or variables [21,22]. Wood et al [21] conducted a scoping review of social media and internet-based communication use by military families. Their research included 11 papers, identified the most popular social media platforms, and highlighted the challenges and advantages of social media use during military deployment [21]. In another systematic review, Tariq et al [22] examined 14 quantitative studies exploring the link between social media use and family connectedness. They discussed how families are connected through social media and its impact on parent-child relationships and broader family connectedness [22].

Finally, 2 review papers examined parents’ use of social media for health information [10,20]. First, Pretorius et al [20] conducted an integrative review of 12 studies on parents’ motivations and use of social media to obtain information about their children’s health, with attention to race, ethnicity, and region. Second, Frey et al [10] conducted a similar scoping review (N=42) on parents’ motivations, understanding, and evaluation of health information on social media and its consequent impact. Both studies found that parents obtained valuable online health information and received support from like-minded others [10,20]. An important difference is that Pretorius et al [20] focused on differences in motivations and platform preferences by race and region, whereas Frey et al [10] focused more on parents’ perceptions and sentiments toward health information on social media.

This systematic review built on but diverged from the previous reviews by adopting a multidisciplinary perspective, integrating various dimensions, and providing a comprehensive and holistic understanding of insights on social media and parenting. It offers a comprehensive overview of parents’ motivations to seek information and the parenting information available on social media and its impacts.

## Methods

### Literature Search and Selection

To collect relevant papers for this systematic literature review, we adhered to the criteria by Kraus et al [23] for identifying scholarly publications. First, we determined relevant keywords and the search formula. All words related to parenting, such as “parent,” “mother,” “father,” “maternal,” “mom,” “dad,” “paternal,” “pregnancy,” “conception,” “postnatal,” “prenatal,” “family,” “kid,” and “child,” were identified, as well as words related to social media, such as “social media,” “influencer,” “Instagram,” “YouTube,” “vlog,” “Facebook,” “Twitter,” and “TikTok.” We combined these terms using Boolean operators (eg, AND and OR) to form the final search formula: TITLE, ABSTRACT, KEY ([Parent* OR mother* OR father* OR maternal OR m?m* OR dad* OR paternal OR pregnancy OR conception OR postnatal OR prenatal OR family OR kid* OR child*] AND [*fluencer* OR Instagram OR youtube OR? log* OR facebook OR “social media” OR twitter OR TikTok]).

Second, a literature search was conducted using the search formula on the Scopus database. Scopus was chosen for 2 main reasons. First, Scopus is the largest multidisciplinary database for science, technology, medicine, social science, and arts and humanities, which is useful for mapping a smaller and more multidisciplinary research field, such as parenting and social media research [26,27]. Second, the Scopus database provides various document data formats, allowing bibliometric software to process them conveniently. All relevant studies published before June 2023 were identified, resulting in 2600 articles in the initial search. The results were saved in RIS format, and information such as title, abstract, authors, keywords, and references was exported. Moreover, we refined the selected articles. The articles collected in the initial search included various document types written in a variety of languages. To guarantee the quality of the papers included in the data analysis, we only focused on full-length and peer-reviewed articles; therefore, other document types, such as conference proceedings and books, were excluded [23]. Furthermore, considering that English is the most common language of research, we only included papers written in English [23]. After this screening, of the 2600 articles, a total of 1540 (59.23%) remained.

Subsequently, we carefully reviewed the remaining papers’ titles, abstracts, and main texts based on the inclusion and exclusion criteria. The inclusion criterion was papers on how parents search for and consume parenting information on social media. All research methodologies, whether empirical studies or reviews, were considered eligible for inclusion in this comprehensive review. Conversely, papers discussing parental mediation and influencers’ motivations to share parenting content were excluded as they fell outside the scope of this review. This procedure left us with a total sample of 338 articles. To guarantee completeness, a snowball literature search was conducted by reviewing the references in each included study, but no new relevant studies emerged.

### Data Analysis

To map the development of parenting and social media research, we conducted a bibliometric and thematic content analysis.

In the bibliometric analysis, we provided a descriptive overview of the research. On the basis of the authors, journal, reference, and publication time, we depicted the evolution of published studies throughout the years, identified the most prolific journals and authors, and detected the most influential articles and authors. BibExcel was used to extract relevant information and perform data analysis.

In the thematic content analysis, we further explored research developments and trends. A keyword analysis examined titles, keywords, and abstracts to identify frequently used words or phrases. A co-occurrence analysis using VOSviewer visually represented the relationships between keywords, creating a co-occurrence network. These analyses enabled us to pinpoint key research topics related to parenting and social media. On the basis of the identified research topics, we conducted a further in-depth investigation of the studies’ contents to categorize them in relation to the research topics. Each study underwent a comprehensive qualitative analysis, which involved screening based on the scientific domain, primary focus, dependent and independent variables, methodology used, target audience, and social media platform used.

## Results

**Figure 1:**
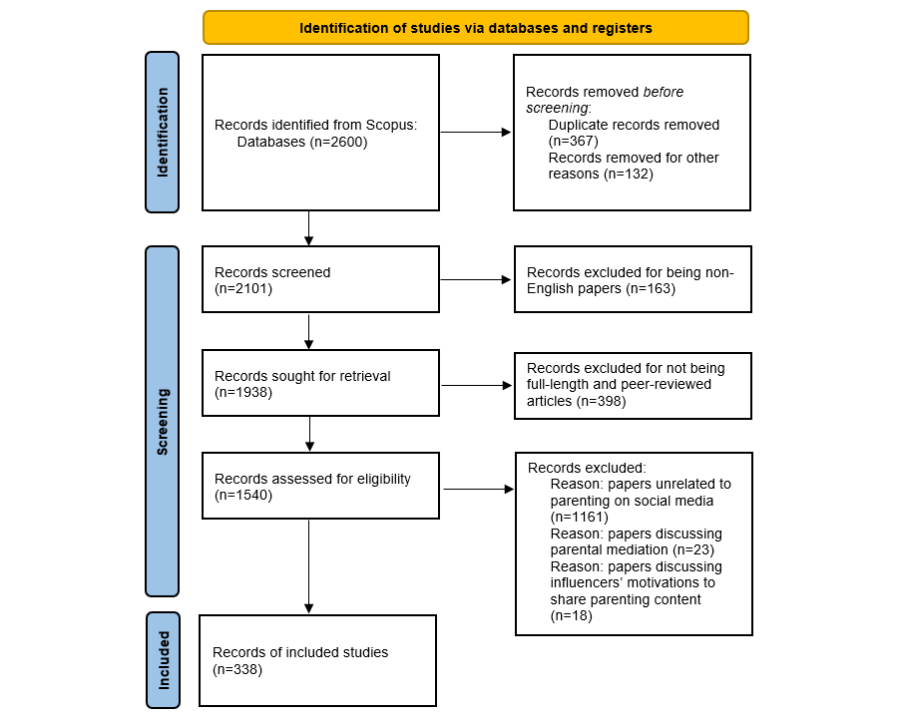
Literature search and refinement criteria for the bibliometric analysis.

Figure 1 presents a detailed overview of the literature search and refinement process.

### Bibliometric Analysis

#### Development of Parenting and Social Media Research

From the graph in Table 1, one can infer that parenting and social media is a relatively emerging research area. The first relevant study was published in 2009. From 2009 to 2014, only 4.4% (15/338) of the papers were published. Conversely, between 2015 and 2022, there was a remarkable surge in the number of publications on parenting and social media. This period encompasses 85.5% (289/338) of the papers analyzed in this study. Moreover, 10.1% (34/338) of the papers were published over the first 5 months of 2023, which indicates that the current general trend of parenting and social media research is rapid growth.

**Table 1:** Number of publications on parenting and social media over the years (n=338).

Year	Publications, n (%)
2009	1 (0.3)
2010	0 (0)
2011	3 (0.9)
2012	2 (0.6)
2013	3 (0.9)
2014	6 (1.8)
2015	10 (3)
2016	11 (3.2)
2017	19 (5.6)
2018	26 (7.7)
2019	33 (9.8)
2020	46 (13.6)
2021	68 (20.1)
2022	76 (22.4)
2023	34 (10.1)

#### Most Prolific Journals and Authors

The articles that we examined were published in 232 different journals, which underscores the broad scholarly attention to the topic of parenting and social media. Of the 232 journals, 13 (5.6%) contributed ≥4 articles, accounting for 20.7% (70/338) of all articles (Table 2). The journal with the most publications related to the topic of parenting and social media was *JMIR Pediatrics and Parenting*, which published 11 articles, followed by *the Journal of Medical Internet Research*, *New Media & Society*, *Feminist Media Studies*, *JMIR Research Protocols*, *Human Vaccines & Immunotherapeutics*, and the *International Journal of Environmental Research and Public Health*. Upon closer examination of the scholarly domains represented by the journals that have disseminated research on parenting and social media, it became apparent that they encompassed a wide spectrum of academic disciplines as classified by Scopus. These disciplines comprised communication, education, medicine, health science, social science, immunology, and allergy. This emphasized the multifaceted research interest in this subject matter.

**Table 2:** Journals that contributed ≥4 articles on parenting and social media research (N=70).

Journal name	Subject matter	Articles n (%)
*JMIR Pediatrics and Parenting*	Technologies, medical devices, apps, engineering, informatics applications for patient and parent education in pediatrics, training and counseling and behavioral interventions, and preventative interventions and clinical care for children and adolescent populations or child-parent dyads [28]	11 (16)
*Journal of Medical Internet Research*	Digital health, data science, health informatics and emerging technologies for health, medicine, and biomedical research [29]	9 (13)
*New Media & Society*	Communication; sociology and political science [30]	6 (9)
*International Journal of Environmental Research and Public Health*	Global health, health care sciences, behavioral and mental health, infectious diseases, chronic diseases and disease prevention, exercise and health-related quality of life, environmental health, and environmental sciences	5 (7)
*Human Vaccines & Immunotherapeutics*	Vaccinology and immunotherapy [31]	5 (7)
*Feminist Media Studies*	Feminist approaches to the field of media and communication studies, with attention to historical, philosophical, cultural, social, political, and economic dimensions and analysis [32]	5 (7)
*JMIR Research Protocols*	Medical and health-related research and technology innovations [33]	5 (7)
*PLOS ONE*	General biochemistry, genetics, and molecular biology [34]	4 (6)
*BMC Public Health*	Focus on the social determinants of health; the environmental, behavioral, and occupational correlates of health and disease; and the impact of health policies, practices, and interventions on the community [35]	4 (6)
*BMC Pediatrics*	Health care in neonates, children, and adolescents, as well as related molecular genetics, pathophysiology, and epidemiology [36]	4 (6)
*Family Relations*	Central focus on families within a wide range of topics of interest to both scholars and practitioners, such as child and parent relationships, cross-cultural and international issues that impact families, family health, family interventions, family life education, and much more [37]	4 (6)
*Health Communication*	Provider-patient (or family) interaction, health campaigns, health information, health promotion, interviewing, health public relations, and gerontological concerns [38]	4 (6)
*Journal of Child and Family Studies*	Behavioral health and well-being of children, adolescents, and their families [39]	4 (6)

Moreover, a total of 1447 different authors made contributions to the development of parenting and social media research. Of the 1447 authors, 1342 (92.74%) published only 1 study on parenting and social media, whereas the remaining 105 (7.26%) published at least 2 papers included in our sample. Scheibling (5 articles) published the most parenting and social media studies in the sample, followed by Cino, Moreno, and Evans, each with 4 publications (Table 3). All other authors in Table 3 published 3 of the studies each. These results indicate that there is no dominant author on the topic of parenting and social media. However, examining the academic collaborations among these authors revealed that many of them share strong academic relationships. For example, Klein and Gonzalez-Hernandez coauthored 4 studies. Buller, Walkosz, Berteletti, Pagoto, Bibeau, Baker, Hillhouse, and Henry worked together on all their published studies. Regarding authors’ affiliations, we found that authors currently affiliated with the University of Toronto, Università Cattolica del Sacro Cuore, University of Wisconsin–Madison, University of Pennsylvania, and Cedars-Sinai Medical Center contributed the most to parenting and social media research.

**Table 3:** Authors who contributed ≥3 articles on parenting and social media research. This table only considers papers published before June 2023.

Author	Current affiliation	Publications, n (%)
C Scheibling	University of Toronto, Canada	5 (0.01)
D Cino	Università Cattolica del Sacro Cuore, Italy	4 (0.01)
MA Moreno^a^	University of Wisconsin–Madison, United States	4 (0.01)
YN Evans^a^	University of Washington, United States	4 (0.01)
MA Bryan^a^	University of Washington, United States	3 (0.01)
AZ Klein^b^	University of Pennsylvania, United States	3 (0.01)
G Gonzalez-Hernandez^b^	Cedars-Sinai Medical Center, United States	3 (0.01)
DB Buller^c^	Klein Buendel, United States	3 (0.01)
J Berteletti^c^	Klein Buendel, United States	3 (0.01)
BJ Walkosz^c^	Klein Buendel, United States	3 (0.01)
SL Pagoto^c^	University of Connecticut, United States	3 (0.01)
J Bibeau^c^	University of Connecticut, United States	3 (0.01)
K Baker^c^	East Tennessee State University, United States	3 (0.01)
J Hillhouse^c^	East Tennessee State University, United States	3 (0.01)
KL Henry^c^	Colorado State University, United States	3 (0.01)
A Lapointe^d^	Université Laval, Canada	3 (0.01)
V Provencher^d^	Université Laval, Canada	3 (0.01)
S Desroches^d^	Université Laval, Canada	3 (0.01)
A-A Dumas^d^	Université Laval, Canada	3 (0.01)
J Robitaille^d^	Université Laval, Canada	3 (0.01)
S Lemieux^d^	Université Laval, Canada	3 (0.01)
RS Gruver^e^	The Children’s Hospital of Philadelphia, United States	3 (0.01)
S Virudachalam^e^	University of Pennsylvania, United States	3 (0.01)
AG Fiks^e^	University of Pennsylvania, United States	3 (0.01)
CT Bishop-Gilyard^e^	University of Pennsylvania, United States	3 (0.01)
A Burke-Garcia^f^	NORC^g^ at the University of Chicago, United States	3 (0.01)
KB Wright^f^	George Mason University, United States	3 (0.01)
JA Manganello	University at Albany School of Public Health, United States	3 (0.01)
HK Tabor	Stanford University, United States	3 (0.01)
JR Levi	Boston University, United States	3 (0.01)

^a^These authors have close academic cooperation.

^b^These authors have close academic cooperation.

^c^These authors have close academic cooperation.

^d^These authors have close academic cooperation.

^e^These authors have close academic cooperation.

^f^These authors have close academic cooperation.

^g^NORC: National Opinion Research Center.

#### Most Influential Authors and Publications

Next, we conducted a local and global citation analysis to identify the most influential authors (Table 4) and publications (Table 5) in our sample. The local citation times refer to the number of citations within the sample, whereas the global citation times refer to the number of citations in the Scopus database. Hence, the discrepancy between the global and local citation index refers to the impact that a paper or author has in domains other than parenting and social media research. In addition, the authors’ local *h*-index was explored, which refers to an author’s number of parenting and social media papers (*h*) that have each been cited at least *h* times by other parenting and social media studies. This index provides an insight into both the quantity (in terms of the number of studies in the domain) and quality (in terms of the impact on other scholars) of an author’s parenting and social media publications. To measure the impact (in terms of shares, discussions, and likes) of parenting and social media research concerning society, we used the Altmetrics score. This score provides an insight into the number of mentions in online media, such as Facebook, Mendeley, Twitter (subsequently rebranded X), and Wikipedia.

**Table 4:** The 20 most cited authors in the parenting and social media area. This table only considers papers published before June 2023.

Author	Local citation times, n	Global citation times, n	Local *h*-index	Altmetrics score
AG Fiks^a^	27	84	3	34
RS Gruver^a^	27	84	3	34
S Virudachalam^a^	27	84	3	34
M Gerdes^a^	23	74	2	18
GK Kalra^a^	23	74	2	18
A Lieberman^a^	23	74	2	18
RI Berkowitz^a^	23	74	2	18
TJ Power^a^	23	74	2	18
J Shults^a^	23	74	2	18
AW Suh^a^	23	74	2	18
CT Bishop-Gilyard^a^	23	74	2	18
LK Lopez	20	139	1	10
JM Sullivan^b^	19	155	1	403
MK Bartholomew^b^	19	155	1	403
SJ Schoppe-Sullivan^b^	19	155	1	403
CM Kamp Dush^b^	19	155	1	403
M Glassman^b^	19	155	1	403
I Yang^c^	14	70	1	7
B Baker^c^	14	70	1	7
K Orton-Johnson	13	48	1	16

^a^These authors have close academic cooperation.

^b^These authors have close academic cooperation.

^c^These authors have close academic cooperation.

**Table 5:** The 10 most cited papers regarding parenting and social media. This table only considers papers published before June 2023.

Publication	Local citation times, mean	Local citation times, n	Global citation times, n	Altmetrics score	Research topic
Jorge et al [40]	7	7	13	6	Examining the self-presentations of mummy and family influencers on social media
Moon et al [5]	3	12	47	34	Parental perceptions of the advantages and disadvantages of the internet and social media as sources of parenting and health information regarding their infant
Baker and Yang [41]	2.8	14	70	7	Investigating the critical role of social media in providing social support for mothers’ lives
Archer and Kao [42]	2.4	12	33	9	Discussing both the negative and positive aspects of social media use for new mothers
Orton-Johnson [43]	2.17	13	48	16	Discussing both the liberating and constraining roles of motherhood in the digital terrain
Haslam et al [44]	2	12	48	74	The status of parents’ use of social media and the potential factors that motivate its use for parenting support
Fiks et al [45]	2	12	36	9	The effectiveness of a Facebook peer-group intervention for low-income mothers to foster behaviors that promote healthy infant growth
Pretorius et al [20]	2	8	16	1	The impacts of social media use on parenting
Ouvrein [46]	2	2	11	31	The impact of exposure to mommy influencer content on parental self-efficacy
Bartholomew et al [47]	1.73	19	155	403	The relationship between new parents’ Facebook use and parenting satisfaction, parenting self-efficacy, and parenting stress

Fiks, Gruver, and Virudachalam obtained the highest number of citations within our sample, indicating that they are the most influential scholars in the parenting and social media domain (Table 4). Among the prolific authors identified in Table 4, they also had the highest local *h*-index, which denotes their substantial body of high-caliber publications. Noteworthily, we found that most of the prolific authors (eg, Scheibling, Cino, Moreno, Klein, and Gonzalez-Hernandez) were not yet highly cited authors. A likely explanation for this finding is that they have only become devoted to this research topic in recent years, and thus, their publications have had less time to accumulate citations. For instance, Scheibling, the most prolific author among them, published all of his studies in the past 3 years. Furthermore, the high deviation between local and global citations of Sullivan, Bartholomew, Schoppe-Sullivan, Kamp Dush, and Glassman indicates that their publications have been frequently cited not only within the area of parenting and social media but also by papers in other disciplines. In addition, their work garnered a high Altmetrics score, which indicates that their publications are frequently discussed and shared on the web.

Given that the citation frequency is closely related to the study’s publication time, we compiled a list of the 10 most cited studies based on their average local citation count (Table 5). Among them, the most influential publication in our sample was by Jorge et al [40]. Their work revealed how mummy influencers reconcile motherhood and their career by examining the ways in which they portray parenting and family, work-life balance, and the boundaries for privacy and intimacy. In addition, the studies by Moon et al [5], Baker and Yang [41], Archer and Kao [42], Orton-Johnson [43], Haslam et al [44], and Pretorius et al [20] are also recognized as highly influential papers due to their substantial number of local and global citations. These papers cover a wide spectrum of topics, ranging from the impact of social media use on parental expectations and attitudes [46], the motivations to seek out parenting information on social media [44], and the effectiveness of social media peer-group interventions for promoting healthy infant growth [45]. Noteworthily, the study by Bartholomew et al [47] had the highest Altmetrics score, which indicates that it has received great attention on the web.

### Thematic Analysis

#### Overview

An analysis of the title, keywords, and abstract fields revealed a total of 1599 title words that occurred 3620 times, a total of 894 keywords that occurred 1610 times, and a total of 5442 abstract words that occurred 42,620 times in our sample. All title words, keywords, and abstract words were then manually screened to group words with similar or identical meanings (eg, “blog” and “blogs”). On the basis of the results of the word segmentation, a keyword analysis and co-word analysis were conducted to identify the most prominent research themes in the area of parenting and social media.

#### Keyword Analysis

The keyword analysis aimed to assess the frequency of the words and phrases used within the titles, keywords, and abstracts of the research papers. A consistent pattern emerged in the use of specific words (Table 6). In particular, the terms “social,” “media,” and “social media” were found to be the most frequently used words or phrases. This prevalence was primarily attributed to their status as primary search keywords in our study. Similarly, terms referring to social media such as Facebook, YouTube, blogs, Instagram, Twitter, and the internet were also found to appear frequently.

**Table 6:** The 20 most frequently used words in paper titles, keywords, and abstracts^a^.

Words or phrases			Frequency, n
**Paper titles**			
	“Social”	186	
	“Media”	157	
	“Children”	61	
	“Facebook”	52	
	“Mothers”	45	
	“Parents”	43	
	“Information”	32	
	“Blogs”	31	
	“Pregnancy”	29	
	“Health”	27	
	“Support”	25	
	“Parenting”	23	
	“YouTube”	22	
	“Content”	21	
	“Instagram”	19	
	“Group”	18	
	“Family”	18	
	“Group”	18	
	“Parental”	18	
	“Covid-19”	16	
**Keywords**			
	“Social media”	167	
	“Pregnancy”	31	
	“Facebook”	27	
	“Parenting”	22	
	“Parents”	21	
	“Covid-19”	20	
	“Motherhood”	20	
	“Instagram”	18	
	“Internet”	18	
	“YouTube”	17	
	“Mothers”	16	
	“Blogs”	15	
	“Social support”	14	
	“Sharenting”	10	
	“Pediatrics”	9	
	“Twitter”	8	
	“Health information”	8	
	“Communication”	8	
	“Breastfeeding”	7	
	“Technology”	7	
**Abstract**			
	“Social”	928	
	“Media”	629	
	“Information”	388	
	“Parents”	330	
	“Health”	311	
	“Mothers”	282	
	“Posts”	239	
	“Facebook”	218	
	“Support”	215	
	“Children”	188	
	“Content”	183	
	“Online”	182	
	“Videos”	176	
	“Women”	171	
	“Group”	164	
	“Parenting”	149	
	“Pregnancy”	145	
	“Intervention”	113	
	“Family”	112	
	“Vaccine”	108	

^a^Percentages corresponding to the frequencies of words in paper titles, keywords, and abstracts cannot be provided due to the unavailability of total word counts across the analyzed papers.

Furthermore, it was evident that words associated with familial relationships, including “mothers,” “parents,” “children,” “child,” and “family,” were frequently used, which aligns with their pivotal roles as stakeholders in the realm of parenting. In addition, terms such as “pregnancy,” “parenting,” “motherhood,” “pediatrics,” “Covid-19,” “vaccine,” and “breastfeeding” were found to appear frequently, which indicates that these topics are the core research concerns in the area of parenting and social media. Moreover, terms such as “support,” “health,” “information,” “health information,” and “social support” indicate the multifaceted purposes for which parents use social media. Finally, the prevalence of terms such as “group” and “intervention” within the abstracts highlights the substantial body of literature focused on interventions through social media.

#### The Co-Word Analysis

**Figure 2:**
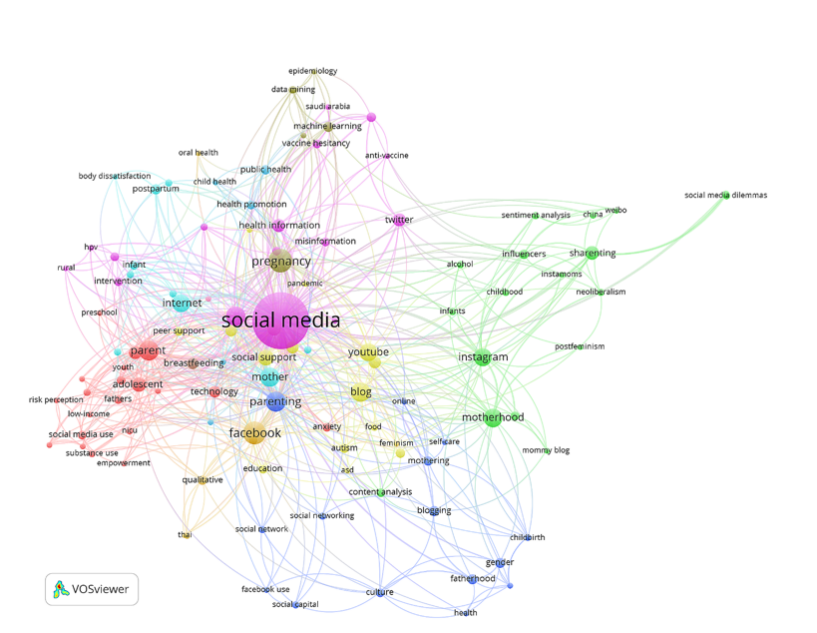
Visualization of the co-word analysis.

The co-word analysis aimed to map the co-occurrence of words that appear in different articles. A visualization of the keywords that often appear together was performed using VOSviewer, and it is depicted in Figure 2. Each node represents an independent keyword, and its size is proportional to the frequency with which the keyword appeared in the articles. The lines between the nodes indicate that the 2 connected keywords appear together in papers, and the thickness of the lines represents the frequency of their co-occurrence.

From Figure 2, one can infer that the nodes for “social media,” “Facebook,” “Instagram,” “YouTube,” “Internet,” “parent,” “parenting,” “mother,” “motherhood,” “pregnancy,” and “adolescent” are larger than those of other keywords, which indicates a strong focus on these topics. The analysis further revealed that all keywords could be grouped into 10 clusters (cf the 10 different node colors in Figure 2), which could be further grouped into 4 thematic research topics through a cluster labeling process.

The first research theme covered studies in clusters 3, 4, and 7. The keywords that reflect the theme were “health,” “health communication,” “health promotion,” “self-care,” “social support,” and “peer support.” Consequently, the first theme was inferred to involve research on parents’ motives to seek information on social media and the identification of variables that may predict the occurrence of this behavior. The second research theme covered studies in clusters 2, 8, 9, and 10, all of which were characterized by keywords closely tied to types of parent-related content on social media and text-mining methodologies—namely, “parenting forum,” “mommy blog,” “instamoms,” “feminism,” “neoliberalism,” “qualitative,” “data mining,” “machine learning,” “natural language processing,” and “sentiment analysis.” This research theme centered on the analysis of online parenting content, where the aim was to construct meaningful insights from such data. The third research theme covered studies in clusters 1 and 6, as indicated by keywords such as “mental health,” “postnatal depression,” “risk perception,” “depression,” “anxiety,” “body image,” and “body dissatisfaction.” These keywords suggest that researchers demonstrated interest in the impact of parenting information on social media. Finally, the fourth research theme covered studies in cluster 5, with keywords such as “intervention,” “obesity prevention,” “anti-vaccine,” “HPV,” and “vaccine hesitancy.” This research focused on parenting interventions with the use of social media.

#### Thematic Content Analysis

##### Overview

To obtain deeper insights into the research content of each of the 4 research themes identified through the co-occurrence analysis, we conducted a thematic content analysis. The first theme consolidated articles that pertained to the underlying motivations of parents to seek information on social media platforms, the second theme encompassed a significant number of articles that explored which parenting-related content can be found on social media, the third theme clustered all articles on the impact of parenting social media information on parents, and the fourth theme contained articles that evaluated interventions on social media. Given that some articles were related to topics across different research themes, we discuss those within the multiple clusters they belong. In the following subsections, we discuss the methodologies and specific social media platforms used in the selected studies. Subsequently, we provide more insights into the various research domains and emphasize the key findings within the extensively investigated research areas.

##### Theme 1: Parental Motivations to Seek Information on Social Media

A total of 14.8% (50/338) of the studies in our sample were found to examine parents’ motivations to seek information on social media. While these studies used various methodologies, half (23/50, 46%) adopted a quantitative approach, primarily applying surveys to gather data. In contrast, the qualitative studies (22/50, 44%) mainly used focus groups, in-depth interviews, and qualitative content analyses to obtain insights into parents’ motivations to use social media as platforms for seeking information. Only a small fraction of the studies (5/50, 10%) used mixed methods research approaches to explore the motivations of parents, mainly through combining surveys, content analysis, or interviews.

In terms of social media platforms, most of the studies within this scope (27/50, 54%) investigated parents’ motivations for seeking information on social media in general rather than specifically focusing on 1 platform. These studies investigated the most popular social media platforms among parents and the motivations that drive them to seek information on social media [48]. Facebook emerged as the most popular platform for parents [48-50]. This is also in line with the keyword and co-occurrence analyses, where Facebook emerged as a big node. Unsurprisingly, studies that examined a specific platform primarily focused on Facebook (16/50, 32% [51,52]), whereas little attention was paid to exploring the motivations of parents on other social media platforms, such as blogs, Twitter, or forums (7/50, 14%).

Parents’ motives to seek information on social media received the most attention in the domain of health information and medicine (34/50, 68%). These studies investigated parents’ motivations for seeking information about general health issues (eg, physical activities), specific diseases (eg, cancer, cleft lip, and autism spectrum disorder), and health-related topics (eg, vaccinations). The 3 dominant motivations for parents to seek health information on social media were as follows: providing and obtaining support from peers, receiving advice and information about one’s child’s diagnosis to guide health decisions, and accessing a community with families who experience the same issues [5,10,50,52-56]. We observed a dominant focus on mothers in the other clusters, whereas the studies within cluster 1 predominantly focused on parents in general regardless of gender.

In addition, attention was paid to parents’ information-seeking motivations within the domains of family science, pregnancy and childbirth, and child feeding (16/50, 32% [57-59]). Such studies did not solely focus on the broader category of parents as one homogeneous group (ie, encompassing both mothers and fathers) but also paid specific attention to mothers and pregnant women individually. Similarly, as in the health-related parenting studies, parents were found to seek informational and emotional support from peers and seek advice and information on various parenting topics, such as pregnancy, activities for their children, and motherhood [44,58,60-63]. However, community feeling was less prominently studied compared with studies in the health and medicine domain.

Across the multiple research subdomains, Moon et al [5] and Suminar et al [58] discovered that mothers attribute greater value to the information they obtain through social media compared with the information they acquire from their family and friends. One of the biggest advantages of social media compared with more traditional sources of parenting information is that information is always available and tailored to parents’ interests and needs [5,20,63,64]. Consequently, young parents in particular exhibit a high level of trust in digital information and opinions provided by other parents on social media [5,44]. Given parents’ great engagement with and active quest for parenting-related support and information on social media, it is evident that these platforms exert a profound impact on their lives and decision-making processes. These subjects are discussed in the following sections.

##### Theme 2: Type of Parenting-Related Content on Social Media

The studies in this cluster (174/338, 51.5%) focused on the types of parenting-related information available on social media. More than half (113/174, 64.9%) of the studies used a qualitative approach, primarily conducting qualitative content analyses to identify the various parent-related topics discussed on social media. Other qualitative methods, such as sentiment analysis, interviews, focus groups, and sociolinguistic analyses, were also applied but on a remarkably smaller scale. The quantitative studies within this cluster (49/174, 28.2%) mainly used quantitative content analysis, whereas a smaller number used surveys to obtain insights into the parenting information available on social media. Finally, some studies (10/174, 5.7%) used a mixed methods approach, mainly combining qualitative and quantitative content analyses to investigate the variety of parenting information. In total, 1.1% (2/174) of the studies were found to have not defined their methodology.

Moreover, to comprehensively analyze the diverse spectrum of parenting-related content on social media, researchers scraped data from various social media platforms. Most of the data in the studies within this cluster were gathered from blogs, YouTube, and Facebook (93/174, 53.4%). A smaller amount of studies (64/174, 36.8%) analyzed parenting content on Instagram or Twitter or examined content from multiple platforms. Other platforms, such as Weibo, online forums, Reddit, TikTok, and WhatsApp, were examined in a minority of studies (17/174, 9.8%). Noteworthily, some studies (11/174, 6.3%) did not explicitly mention or define the specific social media platform from which they gathered their data.

Furthermore, parenting information on social media was studied within a variety of research fields, including health information and medicine, pregnancy and childbirth, family science, nutrition, influencer marketing, and COVID-19. However, most research attention concerning parenting information on social media focused on the fields of health and medicine, followed by the fields of pregnancy and childbirth and family science. In the following paragraphs, we discuss these fields in detail.

First, in the domain of health information and medicine, 2 broad categories were identified—namely, parental health and child health. The studies that focused on parental health encompassed diverse topics, such as medication use, general health practices, alcohol consumption, and infertility [65-67]. The studies that focused on child health information for parents on social media covered a range of health-related topics, such as child vaccinations, autism, child diabetes, cancer, child obesity, sun protection for children, congenital anomalies, elbow fractures, rhizotomies, and mouth sores [68-73]. Across these 2 categories, some studies (14/174, 8%) also examined the quality of and level of trust that parents place in information obtained from social media platforms [72,74,75].

Second, considerable research was conducted in the domain of family science. Such studies primarily focused on the representation and narratives of motherhood and fatherhood on social media [76,77]. Various narratives surrounding the ideals of the “perfect mother” and the notion of a “bad mother” were identified. Other topics, such as the transition into fatherhood, genderfluid parenting, daily life of American families, and grief of parents were detected on social media.

Third, within the domain of pregnancy and childbirth, 3 prominent clusters of information were identified: childbirth (eg, birth stories, hypnobirthing videos, information about miscarriage, and maternal mortality), health and pregnancy (eg, vaccination, physical activities, alcohol, drugs, COVID-19, and anxiety), and representations of pregnancy on social media [78-80]. While research was also conducted in other research domains to map the information about parenting on social media (eg, influencer marketing and nutrition), the volume of such studies was relatively limited.

##### Theme 3: The Role of Parenting Information on Social Media in Parents’ Lives

A total of 21.6% (73/338) of the studies were found to have investigated the relationship between parenting content on social media and parents’ experiences. Most of these studies (41/73, 56%) adopted a quantitative approach, predominantly using surveys to explore the correlations between online parenting information and parents’ daily experiences. Conversely, a smaller subset of studies (17/73, 23%) used qualitative methodologies to delve deeper into parents’ experiences with parenting content on social media. The qualitative methodologies encompassed in-depth interviews, qualitative content analyses, and digital ethnographies. A total of 3% (2/73) of the studies conducted systematic reviews to consolidate existing findings. In addition, 14% (10/73) of the studies used mixed methods research combining various research approaches, such as ethnographies, interviews, focus groups, surveys, content analyses, discourse analyses, and social network analyses. A total of 4% (3/73) of the studies did not clearly specify their methodology.

Regarding the focus of the studies, a substantial portion (38/73, 52%) adopted a broader approach, concentrating on social media in general rather than choosing 1 specific platform. Most of these studies (24/38, 63%) conducted surveys to explore the correlations between parenting content on social media and various dependent variables. These variables include attitudes toward pregnancy and specific diseases, perceptions of social media information, parents’ mental health, and child feeding practices [81]. Among the specific social media platforms, Facebook—particularly specific Facebook groups—was a popular platform to investigate (13/73, 18%). In addition, the studies examined other platforms, such as Instagram, Twitter, YouTube, blogs, or a combination of these (22/73, 30%).

The relationship between parenting content on social media and various variables (eg, parental self-efficacy) was studied within various research domains, such as family science, education, health information and medicine, nutrition, pregnancy and childbirth, advertising, and communication.

First, a notable portion (19/73, 26%) of the studies within this cluster were situated in the domain of family science. They explored a wide range of variables, such as well-being, anxiety, the role of humor in social media posts, the quality and credibility of information, family connectedness, perceived parental skills, and involuntary childlessness. Notably, a range of insights on different topics within the field of family science were identified. For instance, the experimental study by Germic et al [82] revealed that mothers who sought information from online sources had lower perceived self-efficacy than mothers who did not seek online information regardless of the content they were exposed to. Another qualitative study conducted a thematic content analysis and indicated that humor played an important and positive role in reducing parents’ anxieties and distress during the pandemic [83].

Second, a smaller portion (12/73, 16%) of the studies on the role of parenting information in parents’ everyday lives fell within the domain of communication. Most of these studies (9/12, 75%) were conducted in the field of influencer marketing, primarily focusing on “momfluencers.” A momfluencer is a social media influencer (SMI) who has attracted a large number of followers on social media by actively sharing their everyday life of motherhood and who often participates in commercial collaborations [8,84]. More specifically, these studies revealed correlations between the idealized content of momfluencers on Instagram and lower well-being, more anxiety, and less parental self-efficacy among mothers [8,14,84,85]. However, Kirkpatrick and Lee [14] as well as Egmose et al [8] also suggested that momfluencers can have a positive influence by providing support and an online community. Moreover, Ouvrein [46] indicated that a positive relationship exists between regular exposure to momfluencer content and perceived parental self-efficacy for pregnant women. Other studies in the research area of influencer marketing focused more on the promotion of food products or the use and effects of disclosures and visual brand promotion [12,86]. Furthermore, a smaller subset of studies (3/12, 25%) delved into the role of social media in facilitating communication processes between families experiencing military separation or migration [21,87,88].

Finally, some studies (10/73, 14%) were conducted within the research area of pregnancy and childbirth. These examined the correlations between social media use and various aspects of pregnancy, including body satisfaction, childbirth, mental health, and eating disorders. Their findings suggest that social media content that addresses pregnancy-related subjects demonstrates associations with both positive and negative outcomes for pregnant women [89-91]. For instance, the experimental study by Tang et al [89] demonstrated that mothers exposed to body-focused social media posts exhibited higher levels of body dissatisfaction and poorer body image than mothers in the control group. Another study indicated that Facebook serves as a source of social support for new mothers, enabling them to interact with like-minded individuals and feel less isolated during maternity leave by staying in contact with their family and friends [42].

##### Theme 4: Professional Parenting Interventions on Social Media

Given that parents regularly consult social media platforms for parenting information [10], several studies developed professional interventions that targeted parents through various social media platforms. In particular, 12.1% (41/338) of the studies investigated various professional interventions on social media for parents and their children. These interventions were developed and implemented across multiple social media channels, and nearly half (20/41, 49%) were conducted within specific Facebook groups. In 20% (8/41) of the articles, the specific platform used for the intervention was not specified. Other platforms used for implementing the interventions included blogs (5/41, 12%), TikTok (1/41, 2%), WeChat (2/41, 5%), WhatsApp (1/41, 2%), or a combination of these (7/41, 17%).

The professional interventions can be categorized into 3 main research domains, namely, health (20/41, 49%), nutrition (11/41, 27%), and pregnancy (10/41, 24%). First, the interventions within the health domain focused on diverse health topics, such as vaccine concerns, parents of children with specific diseases, parents’ mental health, breast cancer, and sleep in infants. A total of 10% (2/20) of the studies, which addressed parental vaccine concerns and hesitance, indicated that the interventions were effective in improving parents’ attitudes toward vaccination [92,93]. In contrast, a human papillomavirus vaccine intervention by Chodick et al [93] was ineffective at improving the uptake of the vaccine among mothers’ daughters. In addition, the interventions targeting children with specific diseases and their parents (5/20, 25%) were found effective [94]. The target audience of the health interventions varied, encompassing parents, caregivers, mothers, and children.

Second, the interventions in the nutrition domain aimed to enhance parents’ food-related behaviors and decision-making processes. Noteworthily, all interventions that focused on nutrition were specifically tailored to parents, mothers, or caregivers, which is unsurprising given that parents—particularly mothers—are recognized as key influencers in shaping their children’s eating habits [95]. Most of the interventions (30/41, 73%) were highly effective at improving the healthy food decisions that parents made for their children. For example, a peer-group intervention implemented through social media had a significant impact on specific feeding behaviors within families with infants at high risk of obesity [45].

Third, the interventions in the pregnancy domain were designed to improve various aspects, such as anxiety during pregnancy, knowledge about vaccines or pregnancy in general, prenatal stress, maternal mental health, and physical health. All interventions that pertained to pregnancy specifically targeted pregnant women and demonstrated positive outcomes. For instance, the self-help mindfulness intervention by Zhang et al [95] was effective at decreasing prenatal stress and negative affect as well as improving positive affect and mindfulness.

#### Thematic Evolution Analysis

**Figure 3:**
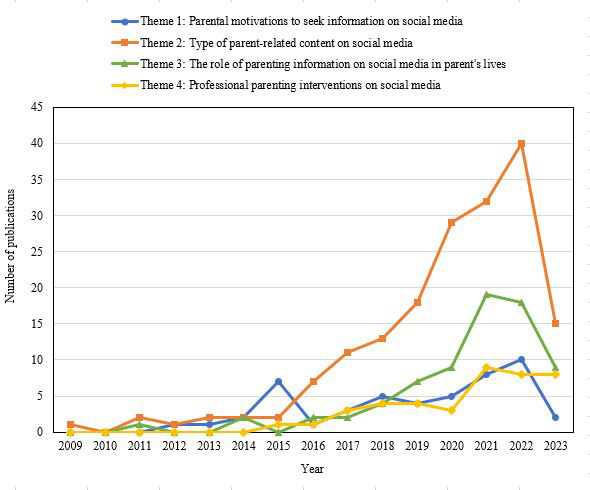
Results of the thematic evolution analysis.

Figure 3 illustrates the thematic evolution analysis that we conducted, offering insights into the progression of the identified themes over time in the realm of parenting and social media research. The analysis revealed notable trends in research focus across different periods. Theme 2, namely, the type of parent-related content on social media, emerged as the initial focal point in 2009 and consistently remained a central area of investigation with the exception of the year 2015. During this period, researchers redirected their attention to understanding the motivations that drive parents to seek information on social media platforms (theme 1). Subsequently, themes 3 and 4 gained prominence, with a growing emphasis on examining the effects of parenting information disseminated through social media channels and evaluating professional interventions.

## Discussion

### Research Gaps and Future Research Recommendations

#### Overview

The area of parenting and social media has received growing academic attention since 2015, aligning with today’s digital turn in information dissemination. In this section, we aim to provide a broader lens for understanding the overall domain of parenting information on social media. Therefore, we discuss the most crucial research gaps that we identified, followed by concrete recommendations for future research to help move our understanding forward of why and how parents consult and are influenced by social media. A summary of recommendations with research questions can be found in Multimedia Appendix 2. These research gaps and recommendations follow the structure of the communication model by Lasswell [96]—communicator, message, medium, audience, and effect—preceded by a brief discussion of the methodologies used in the included studies.

#### Methodologies

Few studies in our sample (28/338, 8.3%) adopted a mixed methods approach to conduct research on parenting information on social media. However, combining qualitative and quantitative research is highly valuable for addressing complex research problems in social sciences [97,98]. Quantitative data reveal the impact of parenting content, whereas qualitative data illuminate individual experiences. Mixed methods are increasingly used within various disciplines, including health sciences, nursing, sociology, psychology, and education [98]. Future research on parenting and social media should use more mixed methods to obtain a holistic understanding of parental interactions and behaviors on social media.

#### Communicator

Within the collected studies, we identified that little to no attention was paid to the sources of parenting content on social media. Crucial to note is that studies that examined the motivations of people to share information on social media were excluded from the sample as they did not align with our objectives. Nevertheless, little to no attention was devoted to answering the question of who is sharing parenting information (eg, everyday parents, medical experts, and parent influencers) on social media and which features characterize these individuals. Hence, numerous questions concerning the source characteristics of individuals sharing parenting information on social media remain unexplored and require further investigation.

Specifically, we noticed that only a fraction of the studies within our sample (13/338, 3.8%) focused on SMIs as sources of parenting information. However, scholars have consistently emphasized that the impact of SMIs on parents and society should not be underestimated [14,99,100]. Specifically, parent influencers provide support and establish a readily accessible and relatable community for parents who want to discuss various parenting topics [100]. Consequently, research has revealed that the content produced by parent influencers exerts a substantial influence on parents in various aspects, including the intention to initiate and sustain breastfeeding and the shaping of parenting ideologies [101-103]. In total, 2 types of momfluencers were distinguished: typical momfluencers who share their personal experiences and professional momfluencers who create content based on their education and professional background [8]. However, research on how parents judge and give meaning to the expertise of information sources on social media is lacking. In addition, while influencer marketing focuses on SMIs’ commercial value, it lacks insights into their role in promoting prosocial behavior [104]. In the context of parent influencers, an important research gap exists in the lack of empirical insights on the effectivity of parent influencers as digital agents for promoting prosocial behavior [105].

#### Message

Studies related to the “message” construct by Lasswell [96] fell under theme 2 (ie, the “types of parenting-related content on social media”). These studies explored the nature of parenting content disseminated on social media, of which a substantial proportion was medical information. Notably, these studies primarily focused on the health information available on social media while often overlooking the assessment of its reliability. Scholars have raised concerns about disinformation on social media [106]. More specifically, one study has argued that there is an absence of gatekeepers for evaluating the information’s veracity before its dissemination; moreover, peers share available information from both professionals and nonprofessionals at an unpredictable speed and pattern, making it difficult to distinguish reliable information [107]. Other research has similarly indicated that misinformation is frequently shared in parenting contexts [69].

When we examined the available parenting content more closely, we noticed that a variety of parenting topics were investigated, such as pregnancy, child vaccinations, nutrition, and specific diseases. This is also reflected in the fact that the studies examined within this scope were published in 232 journals. However, social media content related to parenting styles remained unexplored. Considering that mothers actively seek online parenting information [5,10], it is crucial to gain insights into the different parenting styles that are shared with peers on social media. Furthermore, little attention was paid to the commercial content targeting parents despite evidence showing their vulnerability in early parenthood [3] and the variety of sponsored content on the web [108].

Notably, and similar to the source cluster, little to no research exists on the type of parenting information shared by SMIs. However, momfluencers are extremely popular among pregnant women and first-time mothers, who regularly consult their profiles [8,14,84]. Given the influential voices of SMIs, it is crucial to study both influencer characteristics and the content they share with their large audiences.

#### Medium

This study identified some research gaps in the social media platforms studied. The co-word analysis highlighted “social media,” “Facebook,” and “YouTube” as prominent nodes, indicating a focus on Facebook groups and YouTube videos [109,110]. Instagram and TikTok are 2 increasingly important social media platforms that are currently overlooked. Instagram is highly popular among brand-new mothers, and the second largest group of Instagram users is aged 25 to 34 years [111]; thus, they represent the age demographic of a considerable number of young parents [8]. Similarly, TikTok’s popularity extends beyond young demographics to encompass individuals aged 18 to 34 years, who account for 36% of the platform’s users worldwide [112]. Given that parenting information on TikTok and Instagram remains largely uncharted in research, we recommend that future research endeavors encompass a diverse array of methodological approaches and cover all 4 thematic domains of this study (ie, motivations, content type, impact, and interventions). This multifaceted approach will enable a more comprehensive exploration of these social media platforms.

#### Audience

Most of the studies within this scope (326/338, 96.5%) focused on mothers or parents in general. Although mothers are identified as primary health information seekers and caregivers [113], it is crucial not to overlook the role of fathers. In the 21st century, there has been a discernible increase in fatherly involvement [114,115], as seen in the rise of dad bloggers [76,116]. Dad bloggers attempt to counterbalance the stereotypes and narratives of fatherhood that depict fathers as absent or incapable [116]. Given the scarcity of academic attention to dad bloggers, “dadfluencers,” and fathers in general as a target audience, we recommend that future research explore modern family dynamics in an inclusive manner.

The results of our systematic literature review have already indicated that a substantial portion of research delved into parents’ motivations to seek information on social media (cf cluster 1). Nevertheless, a notable research gap exists in the specific types of online information-seeking behaviors of parents. Furthermore, it would be interesting to investigate whether the types of information seeking are associated with parental consumer behavior as well as parents’ decision-making processes. In addition, research could investigate whether certain types of information lead to more informed parenting decisions.

#### Effects

While there was a substantial number of studies on parenting content on social media (174/338, 51.5%), few examined its impact on parents’ decision-making for their children. Thus, future research is necessary in several key areas. First, neoliberal parenting ideals, which emphasize the individual responsibility of parents and their focus on autonomy [117,118], are increasingly represented on social media [119], but their effects on parenting styles, decisions, norms, and practices remain understudied.

Second, parents devote a significant amount of time to social media seeking support and information [5,10] but face an overload of misinformation on the web [69]. Compounded by their non–“digital native” status, parents often grapple with limited digital knowledge, which necessitates further research on their media and advertising literacy [120]. While current studies predominantly focus on children and adolescents in this regard [121], obtaining insights into parents’ media literacy is equally vital because they not only spend a lot of time on the web but also play a crucial role in their children’s social media literacy [122]. In addition, the overload of health information available both on the web and offline was reported to overwhelm parents, leading to anxiety and confusion [123]. Future research should further investigate these negative effects of information overload on parents in the health domain as well as other domains, such as pregnancy and nutrition.

Third, a nuanced analysis of SMIs’ influence is crucial. Current research mainly emphasizes the negative impact of momfluencers, particularly on mothers [85]. Nevertheless, studies have already indicated that SMIs could in general be interesting sources for promoting prosocial behavior [124,125]. Thus, leveraging momfluencers as allies to endorse prosocial behavior, such as advocating for healthy nutrition, is a highly interesting avenue for future research. However, it is imperative to exercise caution concerning the potential drawbacks associated with the commercial arrangements in which momfluencers engage. Richins and Chaplin [125] demonstrated that parents who seek to ensure their children’s happiness through materialistic parenting inadvertently contribute to the development of materialistic adults in the future. Therefore, future research should scrutinize whether the commercial partnerships of momfluencers inadvertently promote materialistic parenting styles and propose strategies to mitigate such effects.

### Conclusions

While parents easily find their way to social media for parenting information and questions, academic research in this area remains fragmented across diverse disciplines and is still in its infancy. This paper has presented the first study to use bibliometric and thematic content analyses to provide a systematic overview of parenting and social media research. This will enable researchers to have a general understanding of the current state of the art regarding research on parenting information on social media. Current research can be classified into the following 4 main categories: parents’ motivations to seek parenting information, types of parenting-related content on social media, the role of parenting information on social media in parents’ lives, and professional interventions for parents on social media. Insights from the thematic content analysis of these themes helped us identify research gaps and provide recommendations for future research.

However, this study has some limitations. One important limitation pertains to the keywords used to select relevant papers. While we included a comprehensive set of keywords related to parenting, such as “parent,” “mother,” “father,” “maternal,” “mom,” “dad,” “paternal,” “pregnancy,” “conception,” “postnatal,” “prenatal,” “family,” “kid,” and “child,” we did not initially include the terms “infant,” “baby,” or “foetus” in our search formula. Therefore, it is important to acknowledge that the absence of certain keywords in our search strategy may have impacted the inclusivity of our review. Another limitation pertains to the database’s ability to scrape articles. In this study, we only searched for articles in 1 database, namely, Scopus. Even though it is the largest multidisciplinary database of science, technology, medicine, social science, and arts and humanities, we might have missed articles relevant to our topic.

Despite its limitations, this review has significant theoretical and practical implications. First, it highlights the broad interest among researchers from various disciplines, including vaccinations, dietary choices, and pregnancy, in understanding what information can be found for parents on social media and how it affects them. However, the dispersed nature of this research area poses a significant challenge, which could potentially lead researchers to overlook valuable insights from other domains. Therefore, this review serves as a clarion call for researchers to exert a concerted effort to synthesize knowledge across and within domains. Second, this review underscores the significant growth in research pertaining to parenting information disseminated through social media over the past 7 years. The rapid expansion in this field indicates its dynamic nature. As such, this review establishes a robust foundation on which researchers can build to further explore this emerging domain. In addition, we provided an extensive list of future research directions with concrete research questions. By providing this extensive list of research avenues, we aim to encourage future researchers to make substantial contributions and enrich the field’s knowledge base. As for the practical implications, this review highlighted that there is an overload of information for parents on social media. The plethora of (often conflicting) information is often perceived as overwhelming for parents [126]. Therefore, it is crucial to equip parents with strategies for navigating the deluge of information effectively and empower them with the skills to discern and use information to their advantage.

## Appendix

Multimedia Appendix 1 PRISMA (Preferred Reporting Items for Systematic Reviews and Meta-Analyses) checklist.

Multimedia Appendix 2 Future research directions with concrete research questions.

## Abbreviations

SMI: social media influencer
