# Supplementary material for: Parenting Information on Social Media: Systematic Literature Review
Source: JMIR Pediatr Parent. 2024 Oct 23;7:e55372. doi: 10.2196/55372 (PMC11541157; doi:10.2196/55372)
Supplement: Multimedia Appendix 2 [file pediatrics_v7i1e55372_app2.docx]

## Appendix 1

Future research directions with concrete research questions

| Future research directions | Research questions |
| --- | --- |
| **Communicator** |  |
| Sources of parenting content | - Who shares parenting information on social media and what are their characteristics? - How does the nature of parenting content vary depending on the source (e.g., everyday parents, medical experts, parent influencers)? - How do different sources interact with each other on social media? |
| Social media influencers | - How do parents weigh the value of personal lived experiences against scientifically supported content shared by social media influencers? - What meaning do parents give to experts parent influencers vs typical parent influencers? - How do parents rely on social media information next to the more traditional sources of parenting information? - How effective are parent influencers as entertainment educators to promote pro social parenting behaviour? |
| **Message** |  |
| Reliability of information | - How can parents be educated and equipped to critically evaluate and verify the credibility of parenting-related health information circulating on social media platforms? - How do parents perceive and trust parenting information when it is labeled with expert labels? - What are the prevalent themes, sources, and characteristics of misinformation related to parenthood that circulate on social media platforms? |
| Parenting topics | - What types of parenting content on parenting styles is shared on social media and how do they vary across different platforms and user demographics? - What types of sponsored content targeting parents are prevalent on social media, and how do they vary in terms of content, platforms, and targeted demographics? - What ethical implications arise from the presence of sponsored content targeting parents on social media, and how can these be addressed to ensure transparent and responsible marketing practices? |
| Content of social media influencers | - What are the prevalent topics and themes in parenting content shared by social media influencers, and how do they align with the needs and interests of their audience? - How reliable and accurate is the parenting information shared by social media influencers, and what factors contribute to its credibility or lack thereof? |
| **Medium** |  |
| Social media platforms | - Why do parents seek for parenting information on Instagram and TikTok and how do these motivations differ from each other? - What are the prominent parenting-related themes and trends on TikTok and Instagram, and how do they differ from those on other social media platforms like Facebook and YouTube? |
| **Audience** |  |
| Fathers | - How has the evolving role of fathers, including the rise of dad bloggers and dadfluencers, impacted modern family dynamics and parental engagement in caregiving and information-seeking behaviours? - In what ways do dad bloggers and dadfluencers challenge traditional narratives of fatherhood, and how does this influence societal perceptions of parenting roles and responsibilities? |
| Information seeking behaviour | - What specific types of information-seeking behaviours are exhibited by parents on social media, and how do these behaviours vary based on demographics and parental roles? - How does the two-dimensional model of everyday-life information seeking by McKenzie (2003) apply to parental information seeking in today's digital landscape, and how can it be adapted to capture the nuances of online information seeking by parents? - What is the relationship between the type of information seeking behaviour of parents and their consumer behaviour and the decision-making processes? |
| **Effects** |  |
| Neoliberal parenthood | - How do neoliberal parenting ideals influence parenting styles, decision-making, norms, and practices? - What is the impact of internalizing neoliberal parenting ideals from social media on the mental well-being of parents and their perception of their parental role? |
| Misinformation | - What is the media advertising literacy of parents regarding parenting information on social media and how does it affects their parental decisions? - How does information overload affect parental anxiety, decision-making, and overall well-being, and what coping strategies do parents employ to manage this overload? |
| Social media influencers | - What is the influence of parent influencers on parental well-being and decision making processes, considering both the positive and negative impacts? - How can parent influencers be employed in intervention studies to promote healthy nutrition among children? - What is the effect of commercial partnerships of parent influencers on the materialistic parenting styles of parents? |
